# Peer Review of “Raw, Unadulterated African Honey for Ulcer Healing in Leprosy: Protocol for the Honey Experiment on Leprosy Ulcer (HELP) Randomized Controlled Trial”

**DOI:** 10.2196/57310

**Published:** 2024-03-01

**Authors:** Anonymous

**Keywords:** leprosy, ulcers, wounds, honey, neuropathy, nerves, Africa, randomized controlled trial, RCT

*This is the peer-review report for “Raw, Unadulterated African Honey for Ulcer Healing in Leprosy: Protocol for the Honey Experiment on Leprosy Ulcer (HELP) Randomized Controlled Trial.”*

## Round 1 Review

### General Comments

This paper is a protocol description of an important study [1], especially for contexts in which advanced wound care products are often not available. It is a well-written protocol with clear steps to take. Below are some of my feedback; I also included some small textual feedback points in the text. You may not be able to address all the points I raised, as it seems that the trial has already started, but in that case, it would be interesting to describe why or why not in the manuscript’s text.

### Specific Comments

#### Major Comments

1. Please describe why the 84-day cutoff period was chosen.

2. The flowchart is a bit small and thus hard to read.

3. Usually, overlapping inclusion and exclusion criteria are not mentioned.

4. It is not clear why hepatitis B or C were added in the exclusion criteria list.

5. It is not clear for me if patients are clinically admitted or not, and if so, why? For how long? Is this routine care? And what are the discharge criteria?

6. If diabetes is excluded, it may be good to also exclude other known reasons for peripheral neuropathy (eg, vitamin B deficiencies)

7. Are signs of infection also monitored, assessed, or outcome measures?

8. Please explain more on the swabs: what kind of swab is it and what is tested, if this is not part of the research project? In general, it is better to take a routine swab to test for infection (bacterial growth) prior to inclusion instead of prior to randomization, as infection is an exclusion criteria. Also, address this under the heading about “biological specimens.”

9. Explain why the video recording is taking place. It may be interesting to also do it with the last 5 patients if it is performed for monitoring reasons.

10. Why are assessors from Nepal used and not contextual assessors from Nigeria itself (also, is it because of skin color differences of participants in both countries)?

11. Please explain why early analysis is taking place after the inclusion of the first three-eighths of participants.

12. I missed the argument in the discussion that mentioned that honey is often relatively cheap and better available than many advanced wound care products.

13. Include some information about how long data will be stored (number of years), where it will be stored in a secure way, and if it will be shared (pseudonymized) if requested (eg, for reproducibility).

#### Minor Comments

14. Write numbers up to 9 in text.

15. Check abbreviations.

16. Update reference list, include authors, URLs and “assessed on [date]” in references to websites and online documents.

17. Explain the camera used for photography.

18. Please add 2 more references in the discussion.

19. Explain more about the pedometer usage.
